# Clinico‐histopathological and molecular detection of small ruminants’ papillomaviruses in Iran

**DOI:** 10.1002/vms3.1516

**Published:** 2024-07-12

**Authors:** Mohammadreza Ghorani, Hossein Esmaeili, Monireh Khordadmehr

**Affiliations:** ^1^ Department of Pathobiology Faculty of Veterinary Medicine University of Tabriz Tabriz Iran; ^2^ Department of Microbiology and Immunology Faculty of Veterinary Medicine University of Tehran Tehran Iran

**Keywords:** goat, histopathology, papillomavirus, PCR, sheep

## Abstract

**Background:**

Papilloma DNA viruses are one of the viruses that cause skin lesions in ruminants.

**Objectives:**

The clinical, histopathological and molecular characteristics of cutaneous papilloma in ruminants in Iran are to be investigated in this study.

**Methods:**

Samples were collected from 19 small ruminants (5 sheep and 14 goats) with various papillomatosis lesions. The samples taken were studied with histopathological and molecular techniques.

**Results:**

In clinical terms, the lesions appeared in different sizes, ranging from 0.5 to 11 cm, and the cauliflower exophytic masses appeared in other parts of the animal's body. In the limbs, most papilloma lesions have been seen (42.1%). In histopathological examination, perinuclear vacuolation epidermal granule layer with various degrees of hypergranulosis, hyperkeratosis, acanthosis, orthokeratosis and parakeratosis were seen. Moreover, all the suspected samples were positive for papillomavirus using the polymerase chain reaction technique.

**Conclusions:**

Although the prevalence of papillomaviruses in Iranian sheep and goats is low, it seems necessary to distinguish them from other viral skin diseases, such as cutaneous contagious ecthyma, using molecular techniques and histopathology.

## Graphical abstract

In this study, sheep and goats with clinical signs of papillomatosis were identified. Sampling was done from them. The samples were sent for further laboratory tests. Clinical examinations, molecular PCR tests, and histopathological examinations confirmed the presence of papillomavirus. This is the first study of papillomatosis in sheep and goats in Iran.

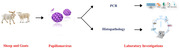

## 1 INTRODUCTION

In the Iranian agricultural economy, small ruminants play an indispensable role and are playing a significant role in the livelihood of nomads and rural farmers (Esmaeili & Hamedi, 2018). In rural flocks, approximately 30% of the goats and sheep are kept nomadic (Esmaeili, Bolourchi, Mokhber‐Dezfouli et al., 2021; Esmaeili, Ghorani, Arani et al., 2021). One of the viral diseases that are sometimes seen in small ruminants is papillomatosis. This disease affects the productivity and economic value of the animals affected, although it may not cause a large number of mortalities. Many animal species and humans have described papillomatosis associated with papillomavirus infection (Zur Hausen, 1996). However, there are few reports of this condition in sheep; in this species, papilloma is usually identified by morphology alone (Al‐Salihi et al., 2020; Smith, 2022).

Papillomaviruses (PVs) are double‐stranded DNA, non‐enveloped and small viruses that infect different species of animals, such as fish, reptiles, birds and mammals (Willemsen et al., 2020). PVs usually have different genera. They are very host‐restricted. In mammals, there have been reports of PV disease in wild and domestic animals and small ruminants (Roperto et al., 2013, 2016; Savini et al., 2016, 2020). There are also known cases of species‐specific PV infection in sheep and goats.

Papilloma lesions probably occur much more frequently in sheep and goats than reported in the literature. They are usually benign and in most cases are self‐limiting. They are diagnosed as warts if noticed, and there is no biopsy or treatment because of a favourable prognosis (Smith, 2022). In a study in which untreated subjects were not controlled, reasonable responses to autogenous vaccines after surgical removal of the most giant warts have been reported. As evidence of an epidemiological origin, the presence of several animals in a single closely confined herd was considered (Smith, 2022). Although rarely has caprine papilloma been found to have a wart virus (Simeone et al., 2008), warts on the udder have a different clinical course and are limited to white goats that have lactated at least once and are of the Saanens or Angoras breed (Smith, 2022). Papillomas occur in the white skin of both the udder and the teat and are usually multiple. They are either flaky or they are shaped like elongated cutaneous horns. Some are transformed into squamous cell carcinomas with a broad base and ulcerated surface. The tumours can rarely metastasize to the supra mammary lymph nodes. Papillomavirus may be involved in the pathogenesis (Manni et al., 1998). Papillomas (warts) of the udder of white goats are a severe problem, particularly in areas with abundant sunshine. An epidemiological survey has shown that the appearance of udder warts is usually seen within a herd 3–6 months after the introduction of an affected goat. It seems to involve exposure to sunlight (Smith, 2022). PVs cause benign and malignant tumours in ruminants, such as cutaneous papilloma, oesophageal, urinary bladder disease, or benign fibroplasia, leading to significant economic losses (Araldi, Melo et al, 2017; Bocaneti et al., 2016). Due to the decrease in milk and meat production and the low value of hides, the incidence of papillomavirus infection may result in significant economic losses for animals. At all ages, ruminant papilloma may occur. Nevertheless, the greater susceptibility is to animals below 2 years of age (Grindatto et al., 2015). By direct or indirect contact, the virus enters the host body via a scratch or injury. In addition, all factors that contribute significantly to the occurrence of this disease include malnutrition, hormone imbalances, artificial insemination, prolonged exposure to sunlight with immunodeficiency, and contamination of food products, including milk machines and syringes (Lindsey et al., 2009). Histopathological features and electron microscopy studies should support clinical lesions on the skin as a significant indicator of primary diagnosis (Araldi, Assaf et al., 2017). Fomites may transmit papillomavirus from animals to each other, including contaminated halters, the nose leads, dairying equipment, grooming and earmarking instruments, rubbing rings, wire fences, or other items that have come into contact with infected ruminants. Transmission of the papillomavirus through sex may also cause genital warts (James MacLachlan, 2016).

The current study highlights the clinical, histopathological and molecular investigations of small ruminant lesions in Iran.

Based on previous experiences, considering the low prevalence of papilloma in Iranian goats and sheep, this study aimed to investigate the signs and diagnosis of papilloma in some small ruminants.

## 2 MATERIALS AND METHODS

### 2.1 History

Macroscopic skin lesions in various parts of the body were studied in 5 sheep and 14 goats in Kurdistan, West Azerbaijan, Isfahan and Tehran provinces, Iran. The fields were not close to each other and they did not have a common pasture. The minimum distance between the herds was 50 km, so they had no contact with each other. All animals had normal health, normal appearance, normal feed and normal body temperature. In three goats and one sheep, papilloma lesions were involved in secondary bacterial infection (Table 1). This secondary bacterial infection did not affect the general health of the animals. The animals involved in the lesions were indigenous breeds (shown in Table 1). Their age was between 5 and 24 months. Fourteen goats and five sheep showed suspicious lesions of papilloma. The disease occurred in both sexes (Table 1). The general condition of the animals was good, and they had no particular problems.

**TABLE 1:** Characterizations of small ruminants infected with papilloma along the locations of the lesions.

Species	Age (month)	Sex	Province	Breed	Secondary infection of the lesion	Head and face	Limb	Under the tail or fat tail	Abdominal wall	Flock size
Sheep	8	Male	Kurdistan	Kurdi	Yes	*				145
Sheep	10	Female	West Azerbaijan	Afshar	No		*			250
Sheep	24	Female	West Azerbaijan	Afshar	No		*			184
Sheep	12	Female	Isfahan	Turki‐Ghashghaei	No			*		365
Sheep	9	Male	Tehran	Zandi	No				*	268
Goat	6	Male	Kurdistan	Markhoz	Yes	*				74
Goat	6	Male	Kurdistan	Markhoz	No	*				420
Goat	8	Male	Kurdistan	Markhoz	No	*				220
Goat	10	Female	Kurdistan	Markhoz	No	*				100
Goat	10	Female	Kurdistan	Markhoz	No			*		67
Goat	13	Female	Kurdistan	Markhoz	No			*		200
Goat	12	Female	West Azerbaijan	Mahabadi	No				*	250
Goat	24	Female	West Azerbaijan	Mahabadi	Yes				*	140
Goat	20	Female	West Azerbaijan	Mahabadi	No		*			132
Goat	9	Male	West Azerbaijan	Mahabadi	Yes		*			210
Goat	14	Female	Isfahan	Turki‐Ghashghaei	No		*			200
Goat	16	Female	Isfahan	Turki‐Ghashghaei	No		*			180
Goat	5	Male	Tehran	Adani	No		*			128
Goat	5	Male	Tehran	Adani	No		*			100

* Presence of lesions in body area of animals.

### 2.2 Sample collection

Sampling was done from animals with papillomatosis lesions. Papillomatosis lesions were sporadically present in the flocks. The animals were healthy and did not suffer from diseases like ecthyma. Suspected tissue samples about 1–2 cm in diameter from skin lesions were fixed in 10% neutral‐buffered formalin, processed routinely, sectioned at 5 µm, stained with common haematoxylin and eosin, and studied by a light microscope for histopathology.

For molecular detection, suspected tissues and scabs were minced by scalpel in phosphate‐buffered saline with antibiotic (streptomycin and penicillin) and stored at −80°C until tested (Ghorani & Esmaeili, 2022).

### 2.3 Molecular detection

Viral DNA was extracted from the samples using a SinaPure Viral Kit (Sinaclon) based on the manufacturer's instructions. We added skin lesions to 1.5 mL microcentrifuge tubes containing 400 µL Lysis solution. We have then 300 µL Precipitation solution and vertexing was added. After that, we transferred the solution to spin columns. We next, centrifuged the tubes at 12,100 × *g*. After that, we added 400 µL Wash buffer I to spin columns and centrifuged at 12,100 × *g*. We washed the spin columns with 400 µL of Wash buffer II centrifugation at 12,100 × *g*. After that, we washed the spin column with 400 µL of Wash buffer II by centrifugation at 12,100 × *g*. Then pre‐heated elution buffer was added in the centre of the columns and incubated for 3–5 min at 55°C. Finally, we centrifuged at 12,100 × *g* for 1 min to elute the nucleic acid. DNA extracted was stored at −20°C until the initiation of molecular testing. We used a single set of polymerase chain reaction (PCR) primers intended to amplify cutaneous PVs according to Forslund et al (1999). The primer sequences are shown in Table 2. The primers were designed for the L1 ORF gene of papillomavirus. The length of the amplified product is 478 bp.

**TABLE 2:** Primes used for the detection of papillomaviruses.

Primer name	Primer sequence (5′ −3′)	Length of amplified product (bp)	Gene	Reference
FAP59	TAACWGTIGGICAYCCWTATT	478 bp	L1 ORF	Forslund et al. (1999)
FAP64	CCWATATCWVHCATITCICCATC			

The 25 µL PCR mixture contained 2.5 µL of DNA extracted, 0.75 µM of each FAP59 and FAP64 primer, 200 µM of each dNTP, and 0.625 U Taq DNA polymerase. PCR was carried out in a thermal cycler (Bio‐Rad) programmed for block temperatures, using the following parameters: 10 min at 94°C and then 45 cycles of 1.5 min at 94°C, 1.5 min at 50°C and 1.5 min at 72°C. We used a 100 bp ladder for gel electrophoresis. Distilled water was used as a negative control. The positive control and the samples were loaded on the electrophoresis gel. Gel electrophoresis analysis of 2% low electroendosmosis agarose for 80 min has been performed on each amplified product. The gel was visualized on a UV transilluminator. On the gel, the bands whose weight is equal to 478 bp are considered positive samples.

## 3 RESULTS

### 3.1 Clinical signs and gross appearance

Nineteen animals were involved in papillomatosis infection from 19 different flocks that were monitored. In each flock, one animal was involved in infection with papillomatosis. Some of the affected animals’ parts were infected with warts. The most common, classical types of papilloma have been identified in these cases and are compatible with ovine and caprine cutaneous lesions.

The lesions were located on the head and face, limb, under the tail and fat‐tail, and abdominal wall. The lesions were variable in size, with dry, horny and verruciform shapes. In all animals, there was no spontaneous recurrence of the lesions, and warts persisted for longer than 5–6 months.

All infected cases (5 sheep and 14 goats) were sporadic in flocks.

According to Table 1, 19 animals (5 sheep and 14 goats) had macroscopic lesions. These lesions were seen on four locations of their bodies (head and face, limb, under the tail or fat‐tail and abdominal wall). Most papilloma lesions were seen in the limbs (8 cases, 42.1%). Other areas involved in lesions were: the head and face (26.3%), under the tail or fat‐tail (15.7%), and abdominal wall (15.7%). Moreover, goats were more infected with the virus than sheep (14 cases). In terms of age, 57.8% of the animals involved in papilloma were under 1 year old, and 42.2% of them were over 1 year old. Also, the highest involvement with infection was seen in females, with 57.8%.

**FIGURE 1:**
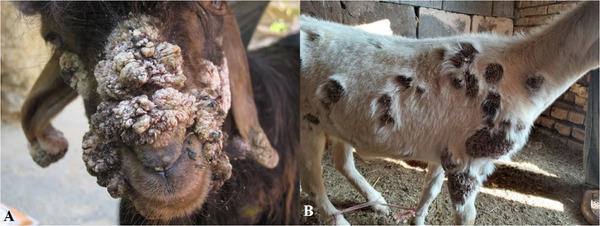
Gross morphology of papillomatosis, goat. Keratinized epidermal proliferations were observed on various body parts, such as the face (A) and abdominal wall (B). There was no ulcer or haemorrhage in the affected parts.

**FIGURE 2:**
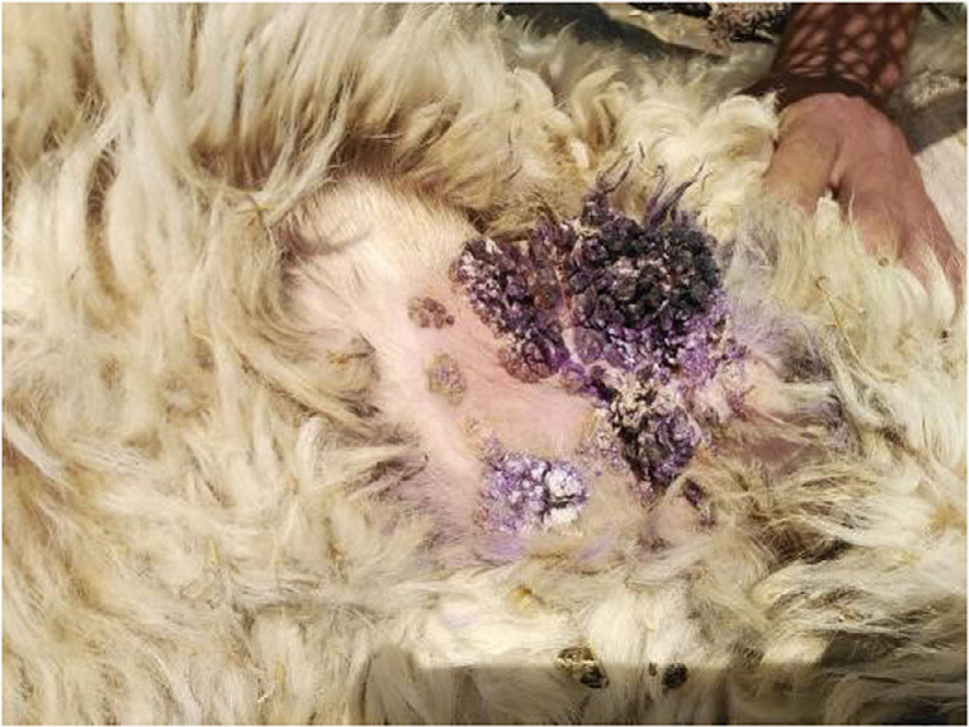
Papillomatous lesions in sheep. Lesions caused by papillomavirus in the area of sheep's abdominal wall.

Here, macroscopic features showed cutaneous papillomatosis, which included elevated and keratinized epidermal proliferations, which occurred on various parts of the body, such as the face, and abdominal wall in goats (Figure 1) and sheep (Figure 2). There were no acute lesions in the affected parts.

### 3.2 Histopathological features

**FIGURE 3:**
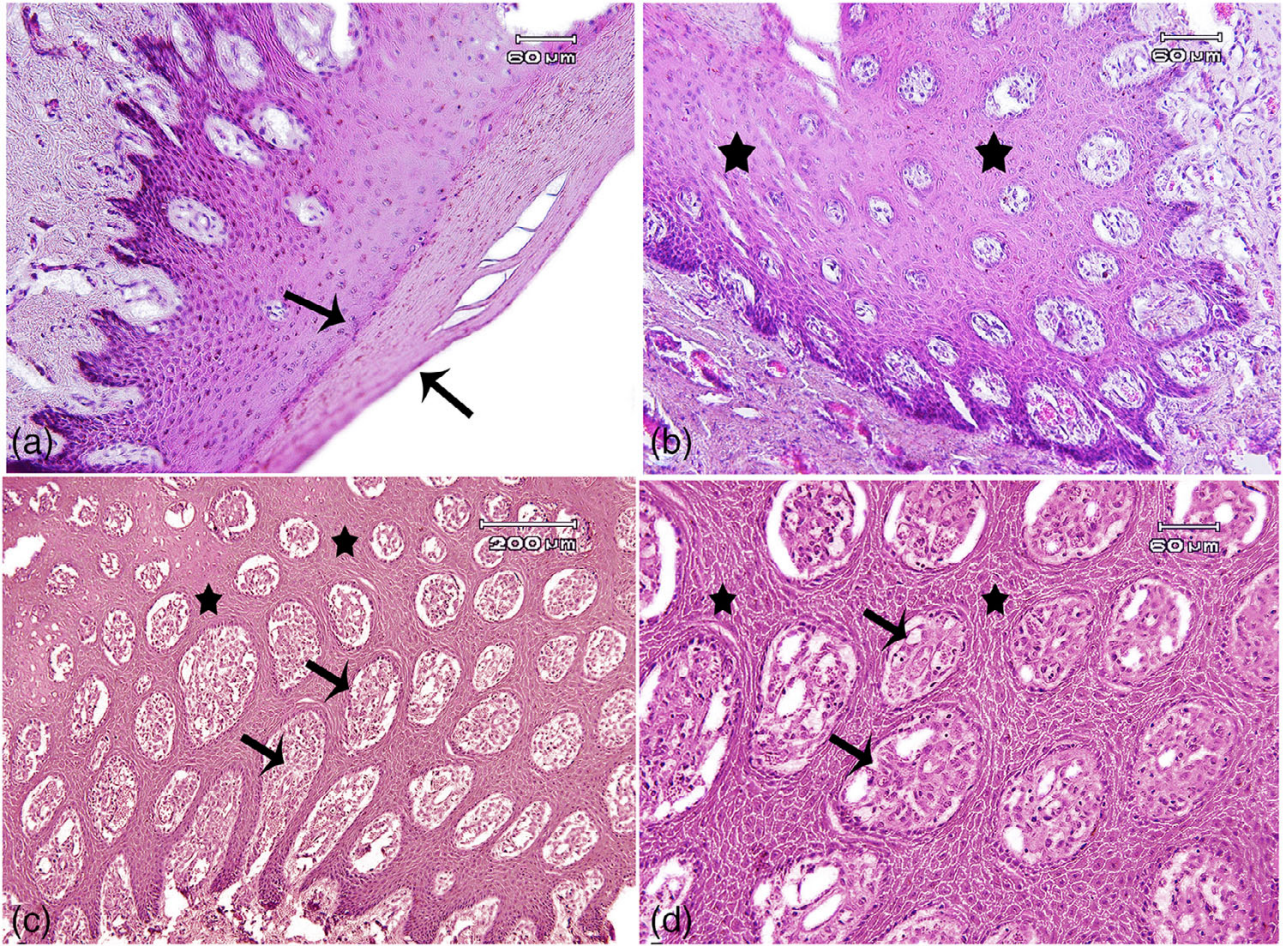
Histopathological features of papillomatosis in the skin, goat. The epidermis shows hyperkeratosis (A: arrows) associated with the proliferation of keratinocytes called acanthosis (B–D: stars) on a core of the dermal stroma (C and D: arrows). There were no acute lesions like ulcer, haemorrhage, and malignancy. haematoxylin and eosin (H&E). D: Higher magnification of Figure C focusing on acanthosis.

Microscopic examinations demonstrated keratinized epidermal proliferation (acanthosis) associated with the core of the dermal stroma. The tumours were characterized by basal cell hyperplasia, mild‐to‐moderate acanthosis (score I to score II), and hyperkeratosis (score I to score II) (Hamada et al., 1990). Those consisted of enhanced thickening of the affected epithelium to gather with the proliferation of the underlying connective tissue. Dermal capillaries were sometimes congested and dilated, associated with mild‐to‐moderate infiltration of inflammatory cells, particularly lymphocytes. There was no malignancy or acute microscopic lesions. The epidermis shows the proliferation of keratinocytes called acanthosis on the core of the dermal stroma (Figure 3A). Figure 3B is a higher magnification of Figure 3A, focusing on the acanthosis.

### 3.3 Polymerase chain reaction (PCR)

**FIGURE 4:**
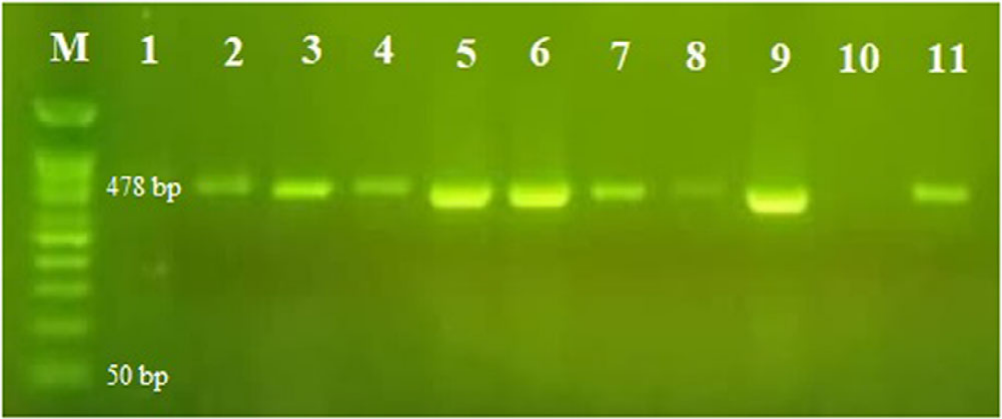
Agarose gel electrophoresis of polymerase chain reaction (PCR) products. The lanes illustrate molecular weight marker 50 bp (M), positive control (9), positive samples (2‐sheep, 3‐sheep, 4‐goat, 5‐goat, 6‐goat, 7‐goat, 8‐goat, 11‐goat) with the 478 bp band, negative sample (1), and negative control (10).

All the suspected samples (19 animals from 19 different flocks) tested by PCR were positive for papilloma (Figure 4).

## 4 DISCUSSION

Despite being ubiquitous, PVs are highly host‐species‐specific (James MacLachlan, 2016). This study confirmed sheep and goat papilloma associated with PVs occur in Iran. The diagnosis of viral papillomatosis was based on the clinical findings, on the histological appearance of the lesions. Epidemiological information about papilloma in sheep and goats is practically non‐existent. The presence of these lesions on the front and lateral aspects of the legs and the muzzle, both areas that are most susceptible to minor trauma during grazing, suggests that this played a role in the transmission of the virus. This mechanism of transmission has been proposed for the transmission of contagious pustular dermatitis (orf) in sheep (Gardiner et al., 1967). Sheep and goat PVs are caused by different strains of the papillomavirus following direct contact with infected animals through scratched skin or other mucosal lesions, as well as passive carriers such as boxes, contaminated food containers, ear tag clips and rectal contact sleeves. There is no effective treatment for papillomatosis. Vaccines did not have any value either. In some cases, the surgical removal of carcinomas can be performed. Prevention includes removing infected sheep and goats from the flocks.

In many animal species, papillomavirus has been associated with several hyperplastic and neoplastic lesions. In addition, papillomas are believed to be precursor lesions for squamous cell carcinomas in goats, cattle and humans. Scientists described papillomas, cornified horns and squamous cell carcinomas on the wool‐free areas of the faces of Merino sheep. They suggested that ovine facial and aural squamous cell carcinomas have a complex aetiology involving unpigmented skin, exposure to sunlight, and infection with papillomavirus (Hamada et al., 1990). Squamous cell carcinoma of the ear has been described in Merino sheep in Patagonia (Uzal et al., 2000).

The clinical investigation of papilloma lesions in small ruminants was conducted in this study. The ages of infected animals were extended between 5 and 24 months. The most anatomical clinical presentation areas of the lesions were the limbs, head and face area, particularly around the eyes and the neck. In some animals, however, these lesions were found in another part of the animal's body. The previous observations reported by another researcher agree with these clinical presentations (Gil da Costa & Medeiros, 2014).

In Turkey, molecular diagnostics and typing of PV have been performed in goats with teat papillomatosis. They reported the first identification of a new putative papillomavirus type (Dogan et al., 2018). In another study, (Simeone et al., 2008), using immunohistochemical and biomolecular techniques, attempted to detect PV in multiple ocular and cutaneous neoplastic lesions spontaneously occurring in two adult Maltese twin goats: one male and one female. Scientists in Romania have developed multiplex PCRs to identify orf and PVs at the same time. Studies have demonstrated that PCR is a fast laboratory method for detecting viral agents that cause severe skin lesions (Strugaru et al., 2012). The papillomavirus lesions in bovine, ovine and caprine animals have been described by Al‐Salihi et al., found in different parts of the affected animals. All lesions show the same or minor differences in histopathological characteristics. Immunohistochemistry revealed positive results in expressing PV antigen and p53 proteins that can be used as diagnostic markers for ruminant papilloma (Al‐Salihi et al., 2020). Odhah et al. have reported a rare case of oral papillomatosis in a goat kid. Contagious ecthyma was ruled out based on the PCR result. History and physical examination findings are beneficial in diagnosing severe clinical cases of papillomatosis (Odhah et al., 2022). A retrospective study of skin samples taken during the cutaneous papillomatosis outbreak in Merino sheep in 1995 was carried out in Argentina. The samples were processed for routine histology, electron microscopy and immunocytochemistry for PVs. The study confirmed the presence of papillomas associated with PVs in sheep in Patagonia (Uzal et al., 2000). In another study, the papillomavirus‐like sequences were confirmed in goat mammary papillomas. This research showed that the papillomavirus probably plays a role in developing precancerous lesions of goat breast skin (Manni et al., 1998). The most sensitive and accurate assay for the detection and quantification of ovine PVs (OaPV) is the droplet digital PCR (ddPCR). This method is even better than real‐time qPCR regarding sensitivity and specificity. DdPCR is a selective molecular diagnostic tool that provides valuable information on OaPV molecular epidemiology and field surveillance (De Falco et al., 2021). For the first time, the highly pathogenic bovine papillomavirus bovine papilloma virus (BPV) was identified and determined in 103 clinically healthy sheep using digital droplet ddPCR with liquid biopsy. In total, with this method, BPVs were detected in 68 blood samples, about 66% of the samples (Roperto et al., 2021). The prevalence of papillomavirus and granulomatous reactions, which occur naturally in sheep in Mosul, Iraq, has been investigated in the Al‐Sabaawy and Al‐Sadi study. These lesions were diagnosed in 3% of examined cases, papilloma and granulomatous infection percentages were 1.23% and 1.84%, respectively (Al‐Sabaawy & Al‐Sadi, 2021).

The findings previously reported have been confirmed by macroscopic presentation and microscopic characteristics of the lesions (da Silva et al., 2015). Although histopathological findings showed marked hyperkeratosis of the epidermis and odd papillary projections into the dermis, many wart lesions appeared as verrucous masses.

Considering that the incidence rate of the disease in sheep and goat flocks is low, if we had access to more samples in more flocks, the results would be more reliable. Because the nature of the disease is sporadic and does not cause severe problems for the health of the animals, usually the farmers do not refer to the veterinarians for the treatment of this disease. For these reasons, in the rural and nomadic breeding systems, papillomatous infections are not actively detected, and there is little access to cases involving papilloma.

Documented research on sheep and goat papillomatosis is limited worldwide, and this aspect, of our study is novel. There is no data in Iran on PVs of small ruminants. This study is the first research on small ruminant papillomatosis.

## 5 CONCLUSION

Based on previous experiences, macroscopic papilloma lesions are not common in sheep and goat populations in Iran. From an epidemiological point of view, considering that PVs are host‐restricted, there is usually no concern about interspecies transmission. However, due to similar skin lesions, papillomatosis should be differentiated from the skin form of contagious ecthyma. Histopathological and molecular tests will help us to make an accurate diagnosis.

Additional studies in other countries are suggested to know the presence of papillomatosis in sheep and goats.

## AUTHOR CONTRIBUTIONS

**Mohammadreza Ghorani**: Writing—original draft preparation; conceptualization; investigation; software. **Hossein Esmaeili**: Data curation; writing—reviewing and editing; supervision. **Monireh Khordadmehr**: Methodology; investigation.

## CONFLICT OF INTEREST STATEMENT

The authors declare no conflicts of interest.

### ETHICS STATEMENT

The authors confirm that the ethical policies of the journal, as noted on the journal's author guidelines page, have been adhered to. No ethical approval was required as this is a review article with no original research data.

### PEER REVIEW

The peer review history for this article is available at https://publons.com/publon/10.1002/vms3.1516.

## Data Availability

The data that support the findings of this study are available from the corresponding author upon reasonable request.
